# Pain Management during Bromelain-Based Enzymatic Debridement (NexoBrid^®^) in a USA Adult Burn Center

**DOI:** 10.3390/ebj5010001

**Published:** 2023-12-19

**Authors:** Martin R. Buta, Domenic Annand, Sarah Findeisen, Sean A. Hickey, Robert L. Sheridan, Jonathan S. Friedstat, John T. Schulz, Branko Bojovic, Edward A. Bittner, Jeremy Goverman

**Affiliations:** 1 Department of Surgery, Massachusetts General Hospital, Boston, MA 02114, USA; mbuta@mgh.harvard.edu (M.R.B.); domenicannand@gmail.com (D.A.); sfindeisen@bwh.harvard.edu (S.F.); sahickey@mgh.harvard.edu (S.A.H.); rsheridan@mgh.harvard.edu (R.L.S.); jfriedstat@mgh.harvard.edu (J.S.F.); jschulz@mgb.org (J.T.S.); bbojovic@mgb.org (B.B.); 2 Harvard Medical School, Boston, MA 02115, USA; ebittner@mgb.org; 3 Sumner Redstone Burn Center, Massachusetts General Hospital, Boston, MA 02114, USA; 4 Department of Anesthesia, Critical Care and Pain Medicine, Massachusetts General Hospital, Boston, MA 02114, USA

**Keywords:** burns, enzymatic debridement, bromelain, NexoBrid, pain management

## Abstract

Outside the United States, bromelain-based enzymatic debridement (BBED) has become an effective tool for the removal of burn eschar. A primary concern with BBED is that it is a painful procedure requiring appropriate analgesia. The purpose of this study was to describe our experience using NexoBrid^®^ (NXB), with a particular focus on pain management. We performed a retrospective review on all 32 adult burn patients enrolled at our institution as part of a multicenter phase 3 clinical trial (DETECT) or the expanded access treatment protocol (NEXT). All patients underwent BBED with NXB of acute deep partial- and full-thickness thermal burn wounds at a major burn center between November 2016 and February 2023. Thirty-two patients with an average age of 42.1 years (SD = 17.4, range 18–72) and an average TBSA of 6.3% (SD = 5.9, range 1–24.5) underwent a total of 33 BBED procedures. Only one patient required an additional NXB treatment, and all patients achieved >95% eschar removal. For pain control during debridement, seven patients required a local block (LB), nine a regional block (RB), and thirteen conscious sedation (CS). Three patients were intubated (INTB) for their burn injury prior to the procedure. There was no statistical difference in Numerical Pain Rating Scale (NPRS) scores during vs. before treatment or after vs. before treatment for all patients or when subdivided by BMI, race, TBSA, total area treated, and anesthetic type (LB, RB, and CS). With appropriate analgesia, the pain associated with BBED of acute deep partial- and full-thickness thermal burns is well tolerated.

## 1. Introduction

Early surgical debridement of burn wounds remains the global standard of care. There have been many notable advances to debridement techniques and tools, especially since the 1950s when a deeper understanding of burn shock and its management resulted in improved survival rates and conditions suitable for safe and more extensive debridement [1,2]. The seminal paper published by Janzekovic in 1970, in which she described tangential excision to sequentially remove burn eschar down to healthy bleeding tissue, demonstrated a technique that markedly improved mortality rates and length of hospitalization [3]. Building on her approach, Tompkins et al. and Herndon et al. solidified the body of evidence supporting early excision with the publication in the 1980s of two pivotal papers that demonstrated improved survival rates and outcomes in patients with major burn injuries [4,5].

While early surgical debridement has demonstrated clear benefits and has become integral to burn care, the accurate estimation of burn depth and the ability for spontaneous wound healing remains a considerable challenge for mixed-depth burn, even in experienced hands [6,7]. The precise and selective removal of eschar and the preservation of healthy tissue is a highly technical process and has particularly important implications for major burns in adults and children and critical areas such as the hands and face [8,9,10,11]. Overly aggressive and imprecise debridement can lead to suboptimal tissue preservation, iatrogenic injury to adjacent healthy tissues, major blood and heat loss, repetitive general anesthesia, impaired function and cosmesis, and overall worse outcomes [12,13,14].

To address the shortcomings of surgical debridement, alternative approaches have been developed, including autolytic debridement, mechanical debridement (e.g., whirlpool, hydrosurgery, wet-to-dry dressings, dermabrasion), biological debridement (e.g., maggot therapy), and laser therapy. None of which has yet proven to be an optimal replacement to the knife [15,16,17]. Enzymatic debridement, another alternative that attempts to resolve the limitations of excisional debridement, has elicited growing interest in recent decades. Most agents, including papain (from papaya) with urea, collagenases from Clostridium histolyticum, and a fibrinolysin–desoxyribonuclease mixture, have proven relatively slow and suboptimal in their efficacy [1,13]. Bromelain-based enzymatic debridement (BBED) agents, derived from pineapple stems, are an effective tool for the removal of burn eschar [13,18,19]. NexoBrid^®^ (NXB) (MediWound, Ltd., Yavne, Israel), a BBED agent that can be applied at the bedside was first approved for clinical application in the European Union (EU) in December 2012 and has since gained considerable traction in burn centers in the EU and received US FDA approval in early January of 2023. Multiple studies have demonstrated NXB’s safety in rapidly, effectively, and selectively removing necrotic eschar from deep partial-thickness (DPT) and full-thickness (FT) burn wounds after a single application, while maximally preserving viable tissue [18,19,20,21,22]. Additionally, compared to traditional tangential excision, BBED was shown to have 50% less blood loss, decreased need for autografting or the amount of autograft required, and significantly better Modified Vancouver Scar Scale (MVSS) scores at 1-year follow-up [10,19,21].

A primary concern with BBED is that it can be a painful procedure without adequate analgesia, potentially a major factor limiting its adoption in routine burn management [23]. Experience with NXB in the U.S. has been gained through a multicenter phase 3 clinical trial (DETECT) and an expanded access treatment protocol (NEXT). In this retrospective study, we describe our experience using NXB with a particular focus on pain management strategies and pain scores.

## 2. Materials and Methods

### 2.1. Data Collection and Statistical Analysis

A retrospective review was conducted on the medical records of 32 adult burn patients, with 9 enrolled in a multicenter phase 3 clinical trial (DETECT Study, ClinicalTrials.gov Identifier: NCT04040660) and 23 enrolled in the expanded access multicenter NEXT trial (NCT04040660), both approved by the Institutional Review Board at Massachusetts General Hospital (MGH) [24]. Both trials adhered to the ethical standards of the World Medical Association’s Helsinki Declaration (adopted in 1964 and amended in 2013). All patients underwent BBED of acute deep partial-thickness and full-thickness thermal burn wounds (TBSA ≤ 30%) at the MGH Sumner Redstone Burn Center between November 2016 and February 2023. Inclusion and exclusion criteria for the two trials are shown in Table 1. Included in this retrospective study were all patients who participated in either trial and underwent BBED at the authors’ study site. Five burn surgeons (S.A.H., R.L.S., J.S.F., J.T.S., and J.G.) were involved in the care of patients included in the study. Data on demographics, burn etiology, and wound and procedural characteristics, including pain management strategies and Numerical Pain Rating Scale (NPRS) scores during debridement and in the 24 h periods before and after debridement, were collected and then analyzed. NPRS scores were primarily recorded by a patient’s nurse in the burn unit according to clinical trial guidelines. The timing and number of pain scores recorded varied for each patient but followed trial protocol.

The Shapiro–Wilk Test was used to test the sample distributions of NPRS scores for normality. The paired t-test was used to compare the changes in NPRS scores for individual patients during the procedure compared to baseline and at 24 h post-procedure compared to baseline. The approach to using NPRS scores essentially treated pre- and post-procedural scores as controls. Statistical analyses were performed using STATA 12 (StataCorp 2011, StataCorp, College Station, TX, USA). Statistical significance was reached at *p* < 0.05.

### 2.2. Anesthesia and Debridement Protocols

Upon admission to the burn center at MGH, patients were examined by a burn surgeon and eligibility for the DETECT or NEXT trials was determined based on study protocol. This review included all subjects who consented to participate in the DETECT trial and were randomized to the NXB treatment arm, whereas all subjects in the NEXT trial were treated with NXB. Subjects for both trials were typically given non-steroidal anti-inflammatory drugs (NSAIDS) and Tylenol for minor burns, and morphine, hydromorphone, oxycodone, gabapentin, or ketorolac for major burns. For procedural pain unrelated to BBED, the standard of care pain management protocol was followed, with patients receiving morphine, hydromorphone, fentanyl, oxycodone, gabapentin, or ketorolac and in some cases, midazolam, dexmedetomidine, lorazepam, or ketamine. Our management of pain during BBED evolved somewhat over the study duration. While no specific protocol was followed, management remained aligned with European consensus guidelines and trial protocol: regional anesthesia for upper or lower extremity burns, local anesthesia for small burns, and analgosedation for the remaining burns [20,25]. Regional anesthesia for the upper extremities included axillary, infraclavicular, and wrist blocks; for the lower extremities, adductor canal saphenous nerve and popliteal fossa sciatic nerve blocks were performed. Local blocks were carried out using lidocaine, bupivacaine, or ropivacaine; regional blocks were conducted using bupivacaine, mepivacaine, or ropivacaine. No liposomal formulations of anesthetics were administrated. Analgosedation was typically achieved using ketamine or IV opiates in combination with benzodiazepines. The goal was moderate sedation during the first 30 min when BBED is most painful. A ketamine 1 mg/kg bolus was usually administered with 5 mg of oxycodone at the start of BBED to provide longer-acting pain control after the ketamine wore off. If needed, ketamine was redosed during the first 30 min of the procedure, however, this was rarely performed. No patients required a redosing of ketamine at the 4 h mark when NXB was removed. Although the entire process of BBED requires a two-hour moist dressing soak before and after the four-hour topical application, the majority of pain occurs during the first 30 min of application of the product. Conscious sedation was therefore not extended beyond this time frame.

Targeted burn wounds were first cleaned mechanically with loose epidermal keratin layers wiped away and blisters removed. Burn wounds were soaked in a hypochlorous acid or Sulfamylon^®^ (Viatris, Canonsburg, PA, USA) solution for at least 2 h prior to treatment and typically overnight. Prior to the application of NXB, a plan for anesthesia was jointly agreed upon and logistics were coordinated by a multidisciplinary team, including the surgeon, anesthesiologist, intensive care nurse, and any other specialists who were part of the care team. For patients already intubated due to burn-related injury or other indications, BBED was carried out as described below. Patient-reported pain scores during debridement and in the 24 h periods before and after debridement were recorded by the nurse in charge of the patient, in coordination with the burn surgeon. For all patients, BBED was carried out within 24 h of burn injury.

Per the manufacturer’s instructions, NXB was then applied to burn wounds in a 2–3 mm thick, even coating and then covered with occlusive sterile dressings. After 4 h, NXB and liquified eschar were removed with a tongue depressor. The wound was then covered with a gauze dressing soaked in Vashe^®^ Wound Solution (Urgo Medical, Fort Worth, TX, USA), 1% Sulfamylon^®^, 3–5% Sulfamylon^®^, 0.9% saline, or sterile water for a 2 h post-debridement soak. After the 2 h soak, dressings were removed, photos of the wound bed were obtained per study protocol, wound depth was assessed, and various dressings were applied per surgeon’s description.

### 2.3. Post-Debridement Care

During the 24 h period after debridement, standard-of-care pain management protocols were followed for all patients. Wounds were assessed daily until discharge and then weekly until wounds had achieved at least 95% closure by epithelialization or graft. After enzymatic debridement, any full-thickness burn wounds or wounds deemed to have a low probability of spontaneous epithelialization and healing were treated with autografts within 3 to 5 days of BBED.

## 3. Results

Thirty-two adult patients (twenty-seven males and five females) underwent BBED between November 2016 and February 2023. Table 2 summarizes patient demographics and burn etiology. Patients had an average age of 42.1 years (SD = 17.4, range 18–72) and were primarily Caucasian (25/32). Flame burns accounted for the majority of burn injuries (22/32), followed by contact burns (5/32) and scald burns (5/32). The average TBSA on admission was 6.3% (SD = 5.9, range 1–24.5).

The anatomic locations of NXB treatment and types of peri-procedural analgesia are presented in Table 3. The average percentage of total surface area of wounds treated with NXB was 4.8 (SD = 3.6, range 1–15). NXB treatment was carried out on 20 upper extremities excluding hands, 16 hands, 12 trunks, 6 buttocks, and 16 lower extremities, with some patients undergoing debridement in more than 1 location. Cases involving more than one target wound for debridement underwent treatment of those wounds concurrently. NXB treatment was judged successful if the procedure resulted in the removal of >95% eschar. Patients underwent a total of 33 NXB treatments as 1 patient required 2 NXB treatments (performed 24 h apart) to achieve adequate eschar removal. All patients achieved >95% eschar removal except for one patient who developed a mild hypersensitivity reaction to NXB which promptly resolved without additional intervention after NXB was removed. This patient was withdrawn from the study. This case was the only adverse event requiring the removal of NXB prior to completion of the 4 h treatment.

**Figure 1:**
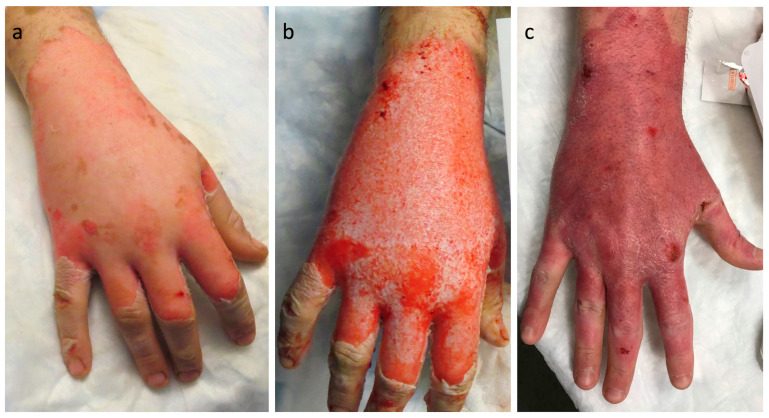
Representative patient admitted with deep partial thickness hot oil scald to right hand. (**a**) Note the pale, non-blanching, dry, overlying eschar to dorsum of hand. (**b**) After 4 h enzymatic debridement with NexoBrid^®^ and 2 h soak, a significant amount of viable dermis is noted. (**c**) Post debridement, the hand was treated with xenograft and silver foam dressing. Silver foam was changed at one-week post debridement and photo demonstrates complete healing at week two.

Pain control during BBED was performed as previously described: seven (22%) patients required a local block (LB), nine (28%) a regional block (RB), and thirteen (41%) conscious sedation (CS, ketamine or opiate + benzodiazepine) (Table 3). No patients required a change in the type of anesthesia administered for failure of adequate pain control. There were no complications noted as a result of the blocks. Three (9%) patients were already intubated (INTB) prior to the procedure, and, except for one patient who was sedated, were able to provide NPRS scores during the peri-procedural period. Following NXB treatment and based on the presence of a deep partial thickness wound, 15 patients (47%) required autografting to ensure wound closure. Only two of the fifteen patients required a single surgical debridement to remove eschar after NXB treatment and prior to autografting. Figure 1 shows a deep-partial thickness hand burn before and after treatment with NXB.

The average NPRS scores 24 h before treatment, during treatment, and 24 h after treatment for the different types of anesthesia were 3.7, 4.4, and 3.6, respectively, and are shown in Table 4. NPRS scores were recorded at variable intervals for each patient with the frequency often depending on a patient’s level of pain. If a patient underwent a regional block, the NPRS score was typically recorded within the first 30 min of treatment. If ketamine was used, the first procedural pain score was typically recorded at least one hour after ketamine administration when the patient was appropriately awake and able to respond to questions about pain control. When subdivided by BMI, race, TBSA, total area treated, and anesthetic type (LB, RB, and CS), there were no statistical differences in the pain scores during vs. before treatment or after vs. before treatment (Table 5).

## 4. Discussion

For many decades, early tangential excision has remained the standard of care to remove burn eschar. Since World War II, various approaches to enzymatic debridement have been developed, though none gained traction in burn centers until NXB was approved by the EU in late 2012. BBED facilitates precise and effective early debridement regardless of burn depth and its diagnosis, removing eschar to accurately reveal a wound’s true burn depth. Compared to tangential excision, with BBED, one is better positioned to visualize and assess a burn wound and select the optimal means for its closure, i.e., epithelialization of intact dermis versus grafting of deeper wounds.

One of the primary benefits of BBED is that it can be performed at the bedside. Many other advantages have been reported. In 2014, in the only published randomized controlled trial on NXB to date, Rosenberg et al. described multiple benefits to using NXB for deep burns compared to standard of care (tangential excisional debridement followed by autografting), including decreased time from injury to complete debridement, decreased need for surgical debridement and autografting, decreased total area grafted, and decreased debridement-related blood loss [26]. Additionally, in children and in hand burns, time to wound closure was significantly shorter with NXB compared to standard of care [26]. Since the EU’s approval of NXB, there has been a growing body of evidence demonstrating the safety, efficacy, and benefits of BBED in treating mixed and full-thickness burns.

Adoption of BBED has been slow in some burn centers due to concerns about procedural pain control [23]. Our study focused on pain management strategies and pain scores for patients undergoing BBED. We compared NPRS scores for adult patients with acute deep partial-thickness and full-thickness burns less than 30% TBSA during NXB debridement and in the 24 h periods before and after debridement. BBED was performed at the bedside within 24 h of injury for all 32 patients, with pain management guided by our center’s standard of care protocol, which accords with the European consensus guidelines [20,25]. In patients with upper or lower extremity burns, a regional block was carried out. Regional blocks can be conducted safely using ultrasound-guided placement, obviating the need for higher risk intubation and sedation. For small burns, such as isolated hand burns, a local block was typically performed. For all other burns, analgosedation was employed, using either ketamine or IV opiates in combination with benzodiazepines (which we classified as CS).

As part of their pain management protocol for BBED, Natalwala et al. reported using gabapentin during and for 5 days after BBED to modulate the pain response, however, in our experience, we achieved acceptable pain control without gabapentin in most cases [27]. For BBED, Galeiras et al. advocate a U-type procedural sedation and analgesia (i.e., conscious sedation), which they used in 17 of 28 patients who underwent BBED for burn wounds [28]. Of the remaining eleven patients, two received local or regional anesthesia and nine required mechanical ventilation. While we did not require epidural anesthesia for any of our patients, it is a useful modality in the types of cases described by Claes et al. In their proposed algorithm for pain management in children and adolescents undergoing BBED, Claes et al. suggest epidural anesthesia for patients with burns to both lower limbs or a single lower limb in which peripheral regional anesthesia cannot be performed due to the wound’s location and associated sensory innervation [29].

Although one would expect relatively good pain scores in patients having any procedure under block or conscious sedation, here we provide additional hard data to support the European consensus guidelines with respect to pain control. We found that NPRS scores were not statistically different during vs. before BBED or after vs. before BBED when patients were grouped by the different types of anesthesia (LB, RB, and CS), BMI, race, TBSA, and total area treated. The average NPRS scores for the different types of anesthesia 24 h before treatment was 3.7, during treatment was 4.4, and 24 h after treatment was 3.6. Depending on the severity of the burn injury, local or regional blocks or IV and oral pain medications were typically sufficient for optimal pain control. More severe and extensive burn injuries usually require general anesthesia, initiated in three patients in this study prior to undergoing BBED. While none of our patients underwent BBED for surface areas greater than 15% (total area treated), per the European consensus guidelines, sequential BBED for large burns is possible for up to 15% TBSA per session. BBED of wounds greater than 15% TBSA per treatment session can be pursued, however, it requires appropriate monitoring and hemodynamic support and is considered an off-label use [20].

While autografting was not a focus of this study, the low percentage of patients who required autografts (47%) is likely attributable to overestimating wound depth prior to NXB treatment. Wound depth was determined subjectively; objective instruments such as laser Doppler imaging were not used.

### Limitations

This study has several limitations. It included a relatively small sample size overall and for each type of anesthesia. A study with a larger sample size may produce different results. Additionally, the study was retrospective in nature and only included adults, thus the results we report may not be entirely applicable to a pediatric population.

The timing of pain scores taken within one of the three peri-procedural periods (24 h before NXB treatment, during NXB treatment, and 24 h after NXB treatment) varied from patient to patient. Given that pain typically peaks during the first 30 min of NXB treatment, patients who did not have pain scores taken during this period were more likely to have lower pain scores, resulting in a selection bias.

Lastly, the study does not compare pain scores and outcomes of patients undergoing BBED with those of a control group of patients undergoing surgical debridement, the gold standard.

## 5. Conclusions

BBED of deep partial- and full-thickness burns is a safe and effective alternative to excisional debridement of burn eschar. Optimal pain management can be achieved during BBED carried at the bedside with careful coordination and planning by care team members and judicious use of analgosedation and local and regional blocks. To increase the adoption of BBED, additional studies are needed that compare anesthesia protocols and short- and long-term outcomes for different types of adult and pediatric burn wounds treated with BBED.

## Figures and Tables

**Table 1:** Inclusion and exclusion criteria for NexoBrid^®^ treatment.

Inclusion criteria—patient level Males and females ≥18 years of age. Thermal burns caused by fire/flame, scalds, or contact. Patient total burns area ≥3% (DETECT Trial) and ≥0.5% (NEXT Trial) deep partial thickness (DPT) and/or full thickness (FT). Patient’s total burns area should be ≤30% TBSA; superficial partial thickness (SPT), DPT, and/or FT in depth. Informed consent can be obtained within 84 h of the burn injury. Patients who are willing and able to sign a written consent form.
Inclusion criteria—wound level At least one wound (a continuous burn area) that is ≥0.5% TBSA (DPT and/or FT) (this minimal wound size should not include face, perineal, or genital area). All planned target wounds (TWs) should meet the following criteria: SPT area that cannot be demarcated from DPT and FT areas should be <50% of the TW’s % TBSA. Wound’s blisters can be removed/ unroofed, as judged by the investigator.
Exclusion criteria—patient level Patients who are unable to follow study procedures and follow-up period. Modified Baux index ≥80. Patients with burned charred fingers, 3rd degree in depth and possibly devoid of circulation. Patients with abraded wound/s that cannot be treated by an enzymatic debrider application (NexoBrid®) will be excluded from the study. Patients with electrical or chemical burns. Patients with circumferential (≥80% of the limb circumference) DPT and/or FT burns are defined as Extremities at Risk (EAR) 2 as described in the protocol. The following pre-enrollment dressings: (a) Flammacerium, (b) silver nitrate (AgNO_3_). Patients with pre-enrolment wounds are covered by eschar heavily saturated with iodine or by SSD pseudoeschar (e.g., pseudoeschar as a result of >12 h SSD treatment). Patients with pre-enrollment escharotomy. Patients with diagnosed infections as described in the study protocol. Diagnosis of smoke inhalation injury. Pregnant women (positive pregnancy test) or nursing mothers. Poorly controlled diabetes mellitus (HbA1c > 11%) in patients with known diabetes as captured in the medical history. BMI greater than 39.0 kg/m^2^ in patients with burn area up to 15% TBSA or BMI greater than 34.0 kg/m^2^ in patients with burn area more than 15% TBSA. ASA greater than 2. Cardio-pulmonary disease (MI within 6 months prior to injury, severe pulmonary hypertension, severe COPD or pre-existing oxygen-dependent pulmonary diseases, severe broncho-pneumonia within 1 month prior to injury, steroid-dependent asthma or uncontrolled asthma). Pre-existing diseases that interfere with circulation (severe peripheral vascular disease, edema, lymphedema, regional lymph nodes, significant varicose veins). Any conditions that would preclude safe participation in the study or add further risk to the basic acute burn trauma (such as severe immuno-compromising diseases, life-threatening trauma, severe pre-existing coagulation disorder, severe cardiovascular disorder, significant pulmonary disorder, significant liver disorder including post-alcoholic abuse impaired function or neoplastic disease, blast injury). Chronic systemic steroid intake. History of allergy and/or known sensitivity to pineapples, papaya, Bromelain, or papain. Current (within 12 months prior to screening) suicide attempt. Mentally incapacitated adults who are incapable of giving legal consent. Enrollment in any investigational drug trial within 4 weeks prior to screening. Current (within 12 months prior to screening) alcohol or drug abuse. Prisoners and incarcerated. Patients who might depend on the clinical study site or investigator.

**Table 2:** Patient demographics and burn etiology.

Total number of patients	32
Gender, No. (%)	
Male	27 (84)
Female	5 (16)
Age, mean (SD, range), years	42.1 (17.4, 18–72)
Ethnicity	
Caucasian	25
Black	3
Asian	1
American Indian or Alaska Native	0
Native Hawaiian or Pacific Islander	0
Other/unavailable	3
TBSA, mean (SD, range) %	6.3 (5.9, 1–24.5)
Burn etiology (N)	
Fire/flame	22
Scald	5
Contact	5

TBSA = total burn surface area.

**Table 3:** NexoBrid^®^ treatment.

Total area treated, mean (SD, range)	4.8 (3.6, 1–15)
Areas treated (can be multiple per patient), No.	
Upper extremities excluding hands	20
Hands	18
Trunk	12
Buttocks	6
Lower extremities	16
Type of peri-procedural analgesia, No. (%)	
Local block	7 (22)
Regional block	9 (28)
Conscious sedation	13 (41)
Intubated	3 (9)
Patients withdrawn from study due to complications, No. (%) *	1 (3)
Patients requiring autograft, No. (%)	15 (47)

NXB = NexoBrid^®^; Total area treated = total area treated with NXB; NPRS = Numerical Pain Rating Scale; * One patient was withdrawn due to hypersensitivity to NXB.

**Table 4:** Peri-procedural NPRS pain scores.

	Mean Peri-Procedural NPRS Pain Scores		
Type of Anesthesia	24 h Period before NXB	During NXB	24 h Period after NXB
Local block	3.7	5.2	4.0
Regional block	4.8	5.3	5.1
Conscious sedation	3.9	4.1	3.7
Intubated *	2.3	3.0	1.8
Mean NPRS pain scores for all types of anesthesia	3.7	4.4	3.6

NXB = NexoBrid^®^; NPRS scores were recorded during debridement and in the 24 h periods before and after debridement; * No NPRS scores were recorded during BBED treatment for 1 intubated patient.

**Table 5:** Analysis of NPRS Pain Scores *.

	Difference in NPRS Pain Scores During vs. 24 h Before Treatment Mean (CI)	Difference in NPRS Pain Scores 24 h After vs. Before Treatment Mean (CI)
Overall (n = 33) **	0.46 (−0.69, 1.61)	0.06 (−0.60, 0.72)
By BMI		
<30 (n = 18)	−0.26 (−2.17, 1.63)	0.33 (−0.63, 1.29)
≥30 (n = 15)	1.28 (−0.16, 2.57)	−0.25 (−1.25, 0.73)
By Race		
White (n = 25)	0.24 (−1.23, 1.72)	−0.18 (−1.00, 0.64)
Non-white (n = 8)	1.11 (−0.54, 2.76)	0.83 (−0.14, 1.79)
By %Total Area Treated		
<5% (n = 21)	0.70 (−0.73, 2.14)	0.17 (−0.48, 0.82)
≥5% (n = 11)	−0.01 (−2.27, 2.25)	0.12 (−1.71, 1.47)
Anesthetic Type ***		
LB (n = 7)	1.02 (−2.01, 4.06)	0.36 (−0.89, 1.61)
RB (n = 9)	0.53 (−1.49, 2.55)	0.37 (−0.84, 1.58)
CS (n = 13)	0.20 (−2.02, 2.42)	−0.20 (−1.36, 0.96)

* NPRS scores were recorded during debridement and in the 24 h periods before and after debridement; ** One patient required 2 consecutive debridements; *** Three intubated patients, not included here, were sedated, and received general anesthesia.

## Data Availability

The data presented in this study are available on request from the corresponding author. The data are not publicly available due to protected database source.
